# Microscopic and statistical evaluation of the marginal defects of composite restorations: in vitro studies

**DOI:** 10.25122/jml-2024-0282

**Published:** 2024-06

**Authors:** Dana Bodnar, Anca Silvia Dumitriu, Ioana Suciu, Bogdan Dimitriu, Mihaela Chirilă, Ruxandra Bartok, Mihai Ciocârdel, Ana Maria Cristina Țâncu, Dan Straja

**Affiliations:** 1 Carol Davila University of Medicine and Pharmacy, Bucharest, Romania; 2 Petroleum - Gas University, Ploiesti, Romania

**Keywords:** composite restoration, stereomicroscope, marginal adaptation

## Abstract

This study aimed to evaluate the quality of dental restorations using laboratory microscopic techniques, focusing on the positioning of restorations relative to cavity edges and identifying common errors, such as incomplete or excessive coronal restorations. A total of 41 extracted lateral teeth, previously treated in vivo with Class I or II composites, were analyzed. Thirty-three of these teeth were sectioned medio-distally or buccal-orally and examined under a research laboratory microscope. Marginal areas were measured using eyepieces with a graduated scale and calibration slide, and a frequency statistical analysis was conducted. The analysis revealed that the teeth had fractious edges, deficient marginal closure, excess composite, incorrectly restored occlusal cusps, and marginal adaptation errors, with approximately half of these errors involving excess material. It was observed that restoration procedures in distal areas are particularly challenging, and clinical errors with excess material occur more frequently than those with a deficit. These findings underscore the need for improved techniques and precision in dental restorations to minimize such errors.

## INTRODUCTION

The quality of composite resin fillings in current dental practice is primarily associated with three key objectives: achieving aesthetic restorations, obtaining functional occlusal contacts to establish and maintain physiological static and dynamic occlusion, and ensuring the marginal sealing quality of the cavity, especially for endodontically treated teeth where the filled root canals are concerned [1-5]. A crucial aspect of high-quality restorations is achieving a smooth surface at the interface between the restoration and the dental surface, devoid of ledges or imperfections [6-9].

Marginal adaptation quality is extremely important in successful restorations. Optimal marginal adaptation, as supported by various authors, entails the absence of microleakage phenomena, such as bacterial penetration from saliva and biofilm formation in the cleavage space between the restorative material and the remaining hard dental tissue [10-13]. The occurrence of marginal infiltration is influenced by several factors, including the size and shape of the cavity, the value of the C-factor, the technique used to insert the restorative material, and the method of photopolymerization [14,15].

In clinical practice and experimental studies, a significant challenge is managing marginal excess and deficits in restorations. Typically, marginal excess is associated with primary restorations and relates to the clinician’s technique in inserting the restorative material into the prepared cavity. Conversely, marginal deficits can be a feature of both primary and secondary restorations.

Addressing these aspects effectively may require an integrated and interdisciplinary approach, particularly in complex clinical cases [16,17]. This approach aims to prevent microleakage and the penetration of salivary biofilm into the interface between restorative materials and dental hard tissues, thereby preventing recurrent or secondary dental caries and their associated complications [12,13].

The purpose of this work was to evaluate the quality of dental restorations using laboratory microscopic techniques, focusing on the marginal adaptation errors of coronal restorations. The study hypothesized that improper insertion techniques contribute significantly to marginal excess and deficits, impacting the overall success of dental restorations. This study aimed to identify common errors and provide data to inform better clinical practices and improve restoration outcomes by analyzing extracted lateral teeth previously treated with composite restorations.

## MATERIAL AND METHODS

The present study involved a microscopic examination of composite resin restorations in extracted posterior teeth with Class I or II cavities. Teeth were either extracted or avulsed due to severe periodontal disease, with cases sourced from private dental offices and the university clinic. Informed patient consent was obtained before extractions for the surgical procedure and the inclusion of extracted teeth as biological samples. In cases of avulsion, patients provided consent for the inclusion of their teeth in the study, and post-avulsion alveolar care was administered in line with surgical norms. Due to the high degree of periodontal compromise and the elapsed time since avulsion, reimplantation was not feasible.

A total of 41 posterior teeth were included in the study. Eight teeth underwent stereomicroscopy alone, deemed sufficient for the intended analysis. Digital photographs were captured using a Leica EZ4D stereomicroscope equipped with a digital camera, employing various lighting techniques (e.g., oblique, transmitted, and reflected light) to highlight specific aspects of interest. For illustrative purposes, only two cases from this group are presented in this paper.

For the remaining 33 teeth, thin cross-sections (medio-distal or buccal-oral) were prepared using a Leica Biosystem Microtome RM2125RTS. These sections were examined for marginal adaptation at medial, distal, buccal, and oral levels using a STEINDORFF POL research laboratory microscope with reflected light. Depending on the cross-section type, only two marginal areas (medial and distal or buccal and oral) were exposed for Class I restorations.The section displaying the largest error identified through stereomicroscopy was selected for further analysis. In cases where marginal adaptation was relatively correct at both margins, one margin was chosen arbitrarily. Digital photographs of each selected marginal area were taken using the microscope’s digital camera.

**Figure 1:**
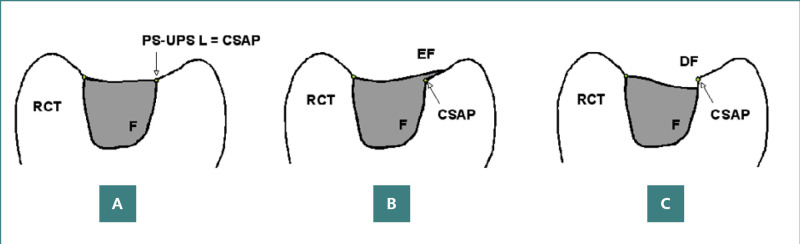
Diagrams illustrating vertical sections of molar crowns with Class I restorations, depicting correct and incorrect restoration placements. A, Correct filling demonstrating optimal marginal adaptation; B, Overextended filling (EF) where the composite material extends beyond the cavosurface angle point (CSAP); C, Underextended filling (DF) where the composite material fails to reach the CSAP. Key: F, filling; RCT, remaining coronal (hard) tissue; PS-UPS L, prepared surface-unprepared surface limit; CSAP, cavosurface angle point.

Fillings were assessed based on their placement at the interface between the prepared cavity surface and the external enamel surface of the tooth (Figure 1 A-C). Ideally, the composite material should align perfectly with the cavity margin, whether beveled or not. Deviations from this ideal included overextended (excess) or underextended (deficit) fillings: overextended fillings (Figure 1B) were characterized by composite resin extending beyond the prepared surface limit onto the unprepared surface, while underextended fillings (Figure 1C) were characterized by insufficient composite resin, failing to cover the entire prepared surface, leaving a portion exposed.

**Figure 2:**
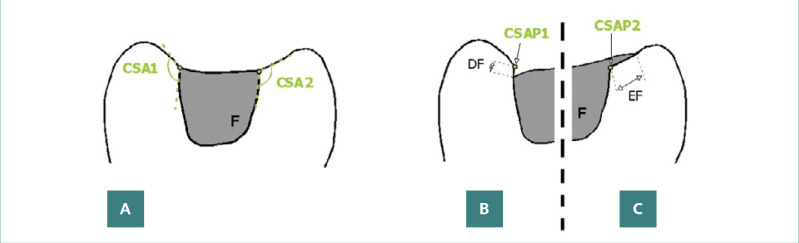
Diagrams of vertical sections through lateral tooth crowns with Class I restorations, illustrating marginal placement accuracy. A, Correct restorations, highlighting the cavosurface angle and its apex; B, Underextended restorations, with measurements indicating the distance from the filling's endpoint on the internal cavity wall to the point it should extend, corresponding to the cavosurface angle tip; C, Overextended restorations, with measurements showing the length of the external crown surface inadvertently covered by the restoration material.

Quantification of marginal adaptation errors was performed using micrometric measurements. The correct restoration is illustrated in Figure 2A. For underextended fillings, the uncovered length from the expected filling margin to the cavosurface angle apex was measured (Figure 2B). For overextended fillings, the excess composite length from the cavosurface angle to the filling's end on the unprepared surface was measured (Figure 2C). These measurements facilitated the analysis of marginal adaptation quality in the restorations.

## RESULTS

Measurements were made using appropriate microscopic eyepieces with reticles and a calibration slide. For calibration, a correct value was attributed to one unit of the eyepiece reticle. This calibration was performed for all microscope objectives planned for the micrometry procedure by aligning the reticle marks with the calibration slide marks, requiring the expertise of a microscopy technician or an experienced microscopist. Dimensional values were read in +/-25µm increments using 40x magnification, as more precise readings cannot be assured at this magnification level. Finally, statistical processing was performed on the measured values, including frequency analyses, to draw conclusions and interpret them in correlation with the clinical experience of the authors. Several consultation sessions with a microscopy specialist were scheduled to structure the work and interpret the results. In this study, two clinical cases of posterior teeth with coronal fillings were examined using stereomicroscopy.

**Figure 3:**
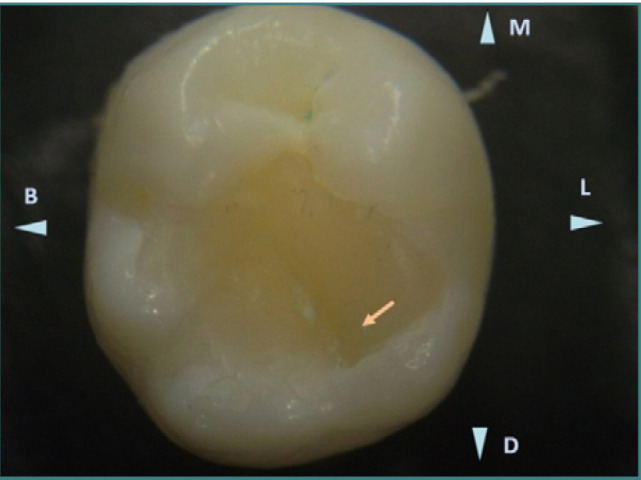
Occlusal view of mandibular third molar (tooth 38) with an extended occlusal composite restoration. The color contrast between the enamel and composite resin allows for clear stereomicroscopic detection of restoration boundaries. The distolingual cusp shows incorrect restoration with a negative relief (ledge) indicated by the arrow. Reflected light stereomicroscopy, magnification 10x. B, buccal; L, lingual; M, mesial; D, distal.

**Figure 4:**
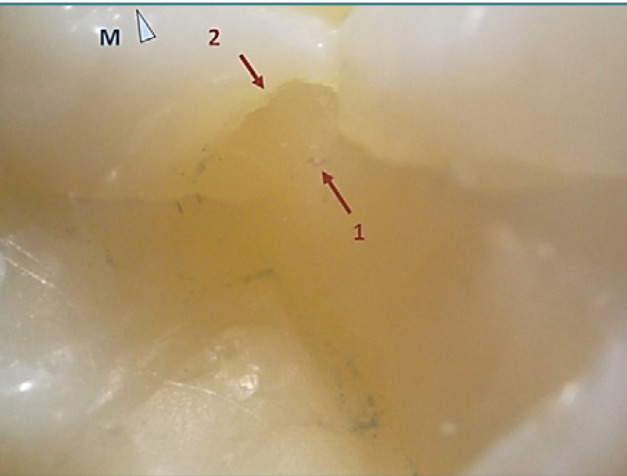
Detailed view of the mesial limit of the occlusal restoration on tooth 38. Arrow 1 highlights accidental fibers embedded in the composite material, while Arrow 2 indicates a ledge on the mesial aspect. Reflected and transmitted light stereomicroscopy, magnification 20x.

The first case is a mandibular third molar, 38, with a defective Class I filling (Figure 3), presenting an extended occlusal composite restoration. Stereomicroscopic examination (10x magnification) revealed distinct color differences between the enamel surface and the composite resin, which allowed for precise detection of the restoration limits. Multiple deficiencies were identified. In the mesial part of the crown, fibers were inadvertently included in the composite material, indicating improper handling (Figure 4, Arrow 1). Additionally, the cavity edge was irregular due to inadequate finishing during the preparation phase, resulting in a poorly defined cavosurface margin (Figure 4, Arrow 2).

**Figure 5:**
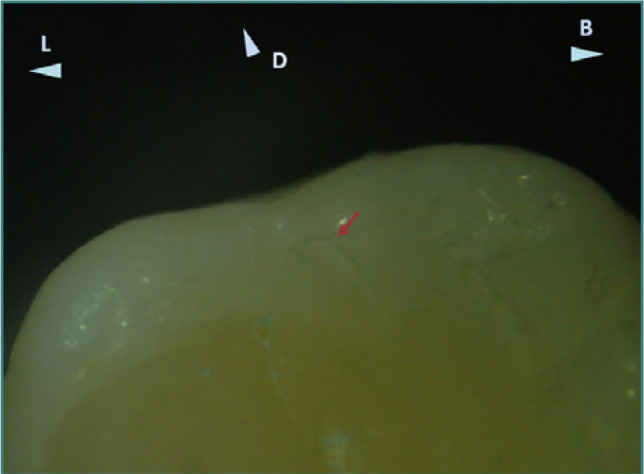
Distal aspect of the occlusal surface of tooth 38, showing a slight excess of restorative material near the distal marginal ridge. Reflected light stereomicroscopy, magnification 20x. B, buccal; L, lingual; D, distal.

**Figure 6:**
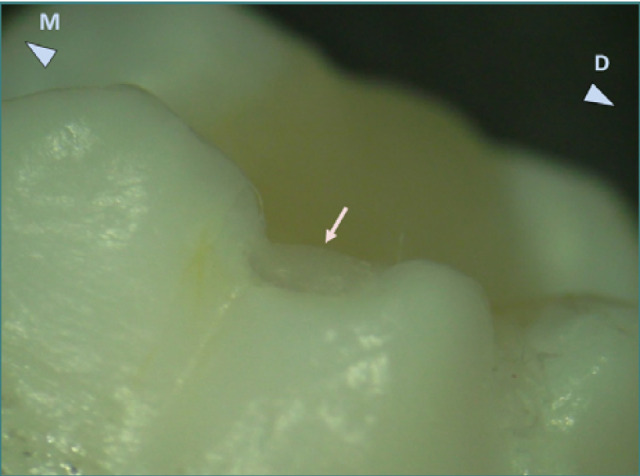
Buccal view of the occlusal composite restoration on tooth 38. Excess composite material is present in the intercuspal groove and is fractured, leaving a small ledge on the distal part. Reflected light stereomicroscopy, magnification 12.5x. M, mesial; D, distal.

The distal part of the crown showed improper restoration of the distolingual cusp’s internal slope (Figure 3). There was also an excess of composite material over the distal marginal ridge (Figure 5), which can contribute to mechanical failures under masticatory forces. In the buccal part of the crown, excess composite material was found covering the intercuspidal groove. This excess was fractured, leaving a ledge on the distal aspect (Figure 6). Such defects suggest contamination and improper handling during the restoration process, compromising the marginal seal and structural integrity of the restoration.

**Figure 7:**
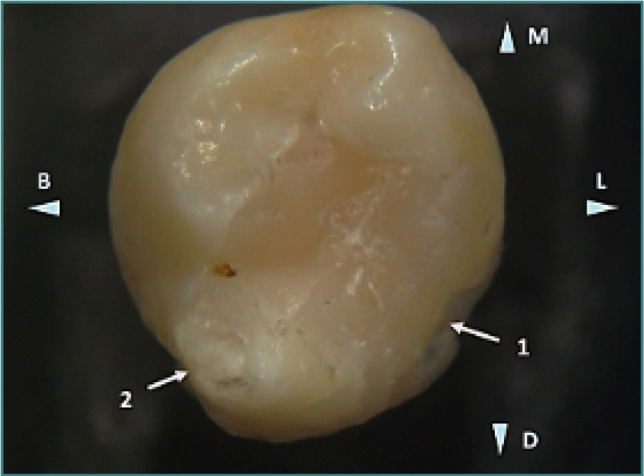
Occlusal image of mandibular second molar (tooth 37) showing a large occlusal restoration that initially restored the distolingual cusp, which is fractured (arrow 1). A fracture is also visible on the distobuccal cusp (arrow 2). Reflected light stereomicroscopy, magnification 10x. B, buccal; L, lingual; M, mesial; D, distal.

The second case focused on a mandibular second molar (tooth 37) with a large occlusal filling extending onto the lingual surface. Initially, this restoration almost completely restored the distolingual cusp. However, stereomicroscopic examination revealed fractures in both distal cusps (Figure 7).

**Figure 8:**
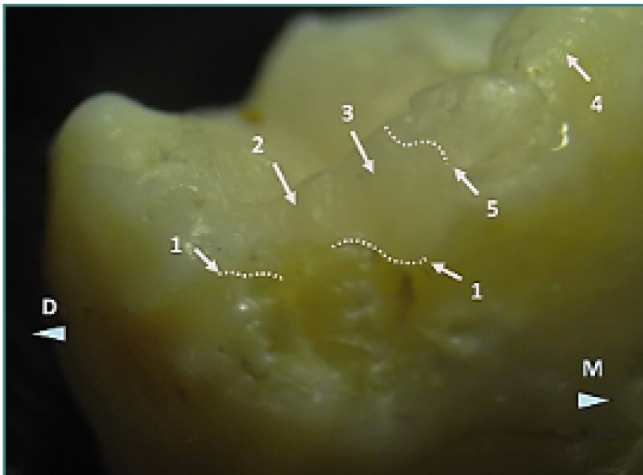
Detailed view of the fracture on the distolingual cusp of tooth 37. Arrow 1 marks the restoration–dental hard tissue limit with a dotted line. Arrow 2 indicates the surface of the fractured restoration, while Arrow 3 shows the unfractured restoration surface. Arrow 4 points to the enamel surface, and Arrow 5 indicates the border between the composite filling and the enamel. Reflected light stereomicroscopy, magnification 20x. M, mesial; D, distal.

**Figure 9:**
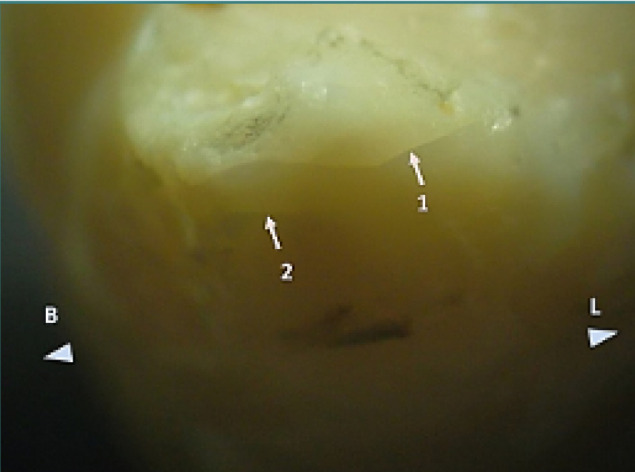
Detailed view of the distobuccal cusp of tooth 37, showing two relatively horizontal cracks at the base (arrows 1 and 2). These cracks, along with the enamel fracture at the cusp's top, suggest occlusal trauma. Reflected light stereomicroscopy, magnification 25x. B, buccal; L, lingual.

Detailed examination of the fractured areas revealed a significant difference in the appearance of the fractured restoration surface compared to the intact restoration (Figure 8). A brown discoloration in the deepest part of the distobuccal cusp indicated dehiscence between the filling material and the enamel wall, contributing to structural weakness (Figure 7). Further fractures were identified at the base of the distolingual cusp (Figure 9), suggesting that occlusal trauma or improper restoration techniques may have caused these fractures.

Restorations involving significant loss of dental hard tissues, such as cusp restorations, require precise cavity preparation and finishing. Unsupported enamel prisms are prone to fracture under mechanical stress, necessitating meticulous restoration techniques. Proper anatomical-layered techniques should be employed for cusp restorations to ensure morphological and functional integrity with optimal marginal closure.

### Estimation of marginal adaptation inaccuracies using micrometric techniques

Micrometric measurements were performed to quantify marginal adaptation inaccuracies. Due to space limitations, representative microscopy images are included to demonstrate the measurement process.

**Figure 10:**
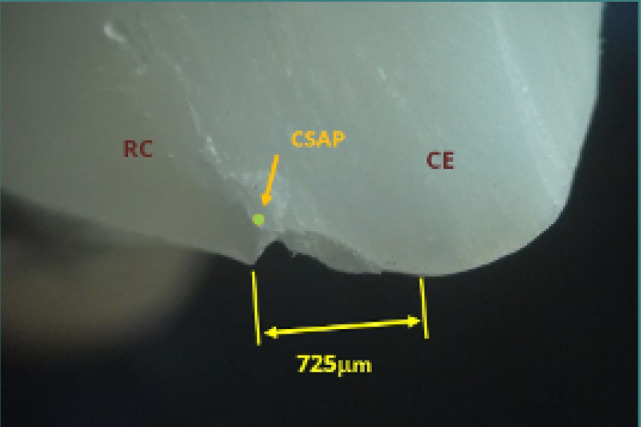
Fractured restoration at the vestibular cusp tip of tooth 15. The overextension amplitude of the composite material could not be evaluated due to the marginal loss of composite material. The position of the point corresponding to the apex of the cavosurface angle is approximately indicated. Reflected light microscopy, magnification 40x.

Figure 10 illustrates a case where part of the cusp was covered with a thin layer of composite without pre-existing marginal beveling. This layer was fractured, preventing precise micrometric assessment of the excess material.

**Figure 11:**
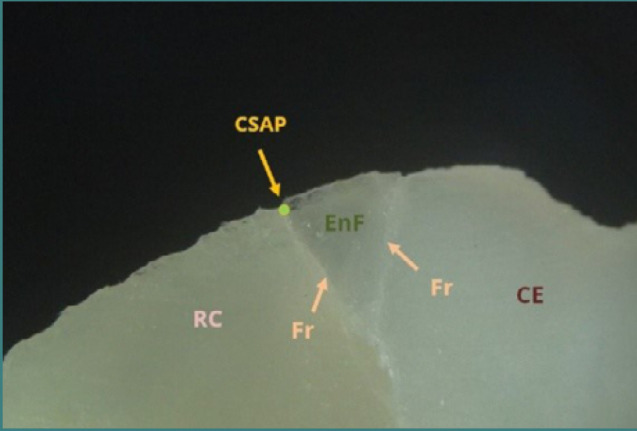
Restoration on tooth 35 showing a marginal enamel fracture near the cavosurface angle. An enamel fragment (EnF) is slightly detached, while no excess or deficit of material is observed. Reflected light microscopy, magnification 40x. EnF, enamel fragment; RC, resin composite; CE, cusp enamel; Fr, fracture; CSAP, cavosurface angle point.

Perfect marginal adaptation was rare. Even seemingly correct restorations often exhibited other defects, such as enamel fractures. Figure 11 shows a marginal enamel fracture in tooth 35, with a slightly detached wedge-shaped enamel fragment. Such fractures can arise from unsupported enamel prisms and occlusal interferences, compromising the restoration's clinical viability.

**Figure 12:**
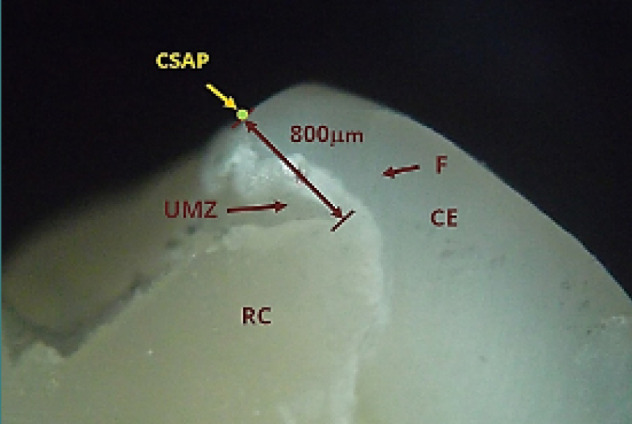
Underextended restoration on tooth 37, showing a vertical fracture of the buccal cusp. The material deficit, measured by micrometry, is approximately 800 µm. Reflected light microscopy, magnification 40x. RC, resin composite; F, fissure; UMZ, undermining zone (filling absent); CE, cusp enamel; CSAP, cavosurface angle point.

Figure 12 presents an underextended restoration where unsupported enamel was left unaddressed, leading to a vertical fracture of the buccal cusp over time. The material deficit was measured at approximately 800 micrometers.

**Figure 13:**
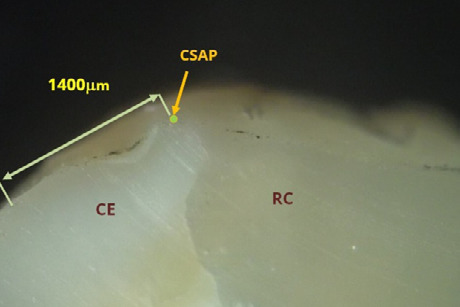
Overextended restoration on tooth 36, with the composite extending over the external surface of the molar without a beveling area. The overextension length is approximately 1400 µm. Reflected light microscopy, magnification 40x. RC, resin composite; CE, cusp enamel; CSAP, cavosurface angle point.

**Figure 14:**
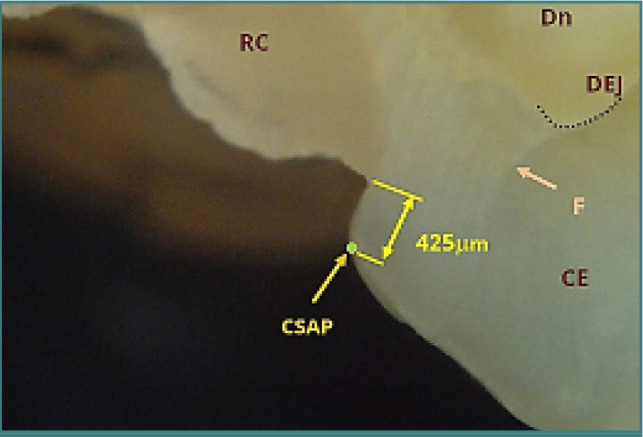
Class II cavity restoration on tooth 26 near the buccal cusp, showing a material deficit of approximately 425 µm. A vertical crack in the enamel (F) extends to the dentin-enamel junction (DEJ). Reflected light microscopy, magnification 40x. CSAP, cavosurface angle point; RC, resin composite; CE, cusp enamel; Dn, dentin; DEJ, dentin-enamel junction.

Figures 13 and 14 depict overextended restorations on non-beveled surfaces. Thin layers of material extended onto the external surface are prone to fracture under masticatory forces. Figure 13 shows an overextended restoration on tooth 36, extending approximately 1400 micrometers. Figure 14 depicts a Class II cavity on tooth 26 with a material deficit near the buccal cusp, measured at approximately 425 micrometers, which can promote secondary caries and occlusal interference.

### Statistical analysis of marginal adaptation deficiencies

Micrometric measurements were taken for 33 teeth sections to assess marginal adaptation deficiencies. The largest deficiency for each tooth was recorded, and in cases with no deficiencies, a section was chosen without predilection. Table 1 lists the number of teeth, types, deviations determined by microscopy (in micrometers), and corresponding figures in the paper.

**Table 1:** Degrees of marginal misfit in composite restorations obtained by micrometry

Tooth no.	1	2	3	4	5	6	7	8	9	10	11
Tooth	MaSM	MxSm	MaFM	MaSm	MxSM	MxFM	MaFM	MxFPm	MaTM	MxFM	MaFPm
Error mag.(mm)	-800	650	1400	-575	0	450	900	675	725	100	-400
Fig.	11	-	12	-	13	-	15	-	16	-	-
Tooth no.	12	13	14	15	16	17	18	19	20	21	22
Tooth	MaTM	MxTM	MxSPm	MaFM	MaSPm	MaFPm	MaSM	MxFPm	MxSM	MxFM	MxSPm
Error mag.(mm)	825	550	675	750	0	600	200	150	500	425	625
Fig.	17	-	18	-	19	-	20	-	-	21	-
Tooth no.	23	24	25	26	27	28	29	30	31	32	33
Tooth	MaTM	MxFM	MaSPm	MaTM	MaTM	MaSM	MaSPm	MxSM	MxFPm	MaSM	MxTM
Error mag. (mm)	1125	900	-50	675	1050	-550	350	225	-175	575	-925
Fig.	-	-	-	-	-	-	-	-	-	-	-

Error mag, error magnitude; MaFPm, mandibular first premolar; MaSPm, mandibular second premolar; MaFM, mandibular first molar; MaSM, mandibular second molar; MaTM, mandibular third molar; MxFPm, maxillary first premolar; MxSPm, maxillary second premolar; MxFM, maxillary first molar; MxSM, maxillary second molar; MxTM, maxillary third molar.

A frequency analysis was conducted on the micrometric values (Table 2). Values were divided into seven class intervals of 335 micrometers, encompassing the maximum negative value (-925 micrometers) and a value close to the maximum positive value (1400 micrometers). Although six intervals are standard, seven were used for greater relevance. Table 2 organizes the values in ascending order, assigning them to respective frequency classes. Table 3 presents the frequency data by class, including interval numbers, limits, frequency, relative frequency, and cumulative relative frequency.

**Table 2:** Classification of composite restoration cases by frequency classes

Tooth number	Tooth	Error mag	Frequency class	Tooth number	Tooth	Error mag	Frequency class
33	MxTM	-925	Cl1	32	MaSM	575	Cl5
1	MaSM	-800	Cl1	17	MaFPm	600	Cl5
4	MxSPm	-575	Cl2	18	MaSPm	600	Cl5
28	MaSM	-550	Cl2	22	MxSPm	625	Cl5
11	MxFPm	-400	Cl2	2	MxSPm	650	Cl5
31	MxFPm	-175	Cl3	8	MxFPm	675	Cl5
25	MaSPm	-50	Cl3	14	MxSPm	675	Cl5
5	MxSM	0	Cl3	26	MaTM	675	Cl5
16	MaSPm	0	Cl3	9	MaTM	725	Cl5
10	MxFM	100	Cl4	15	MaFM	750	Cl5
19	MaFPm	150	Cl4	12	MaTM	825	Cl6
30	MxSM	225	Cl4	7	MxFM	900	Cl6
29	MaSPm	350	Cl4	24	MxSPm	900	Cl6
21	MxFM	425	Cl5	27	MaTM	1050	Cl6
6	MxFM	450	Cl5	23	MaTM	1125	Cl7
20	MxSM	500	Cl5	3	MxFM	1400	Cl7
13	MxTM	550	Cl5				

Error mag, error magnitude

**Table 3:** Frequency data of marginal misfit measurements in composite restorations

Interval number	Lower interval limit	Upper interval limit	Frequency	Relative frequency	Cumulative frequency
1	-925	-590	2	6,06	6,06
2	-590	-255	3	9,09	15,15
3	-255	80	4	12,12	27,27
4	80	415	4	12,12	39,39
5	415	750	14	42,42	81,82
6	750	1085	4	12,12	93,94
7	1085	1420	2	6,06	100,00

**Figure 15:**
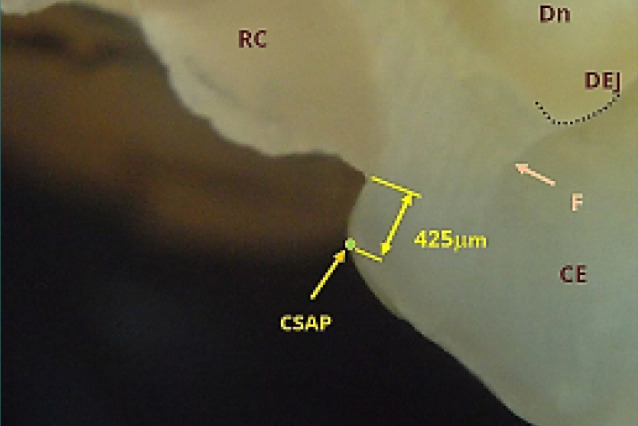
Histogram and frequency curve for the degree of marginal lack of adaptation obtained using micrometry. EM, error magnitude.

**Figure 16:**
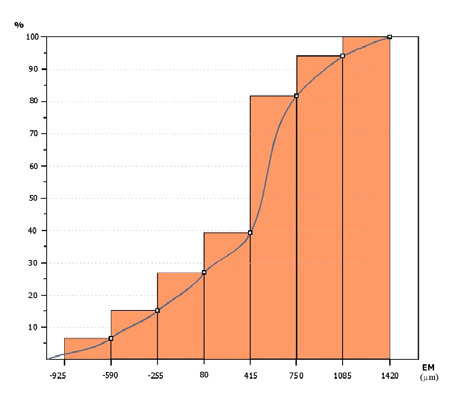
Cumulative frequency graph and cumulative curve for the degree of marginal lack of adaptation obtained using micrometry. EM, error magnitude.

Figure 15 displays the histogram and frequency curve, illustrating the distribution of marginal adaptation deficiencies, while Figure 16 shows the cumulative frequency graph and curve, representing the summed frequencies for each class interval. The histogram provides a visual depiction of data distribution, while the cumulative frequency graph illustrates the overall distribution pattern.

## DISCUSSION

The main outcomes of this study indicate that marginal misfit is a significant issue in composite restorations, with common errors including overextensions and deficits. Through microscopic analysis of extracted teeth, we were able to quantify these errors and identify their causes, offering insights into improving clinical practices. Microscopic studies on extracted teeth allow for a thorough evaluation of filling quality [18-22]. Although these studies have a scientific character, they reveal common errors in restorative procedures, the causes of filling failures, and potential methods to avoid such inaccuracies. Based on extensive microscopic studies, we have determined that laboratory stereomicroscopy and in vivo microscopy using endodontic microscopes enable detailed assessment of dental morphology and aesthetics, while transmitted or reflected light laboratory microscopy provides a precise evaluation of marginal adaptation and endodontic sealing quality [23-25].

Excess or deficient composite material influences marginal fit and longevity of restorations [26-29]. Ideally, microscopic evaluation of marginal adaptation should be performed on cross-sectional surfaces, not just exterior stereomicroscopic evaluation. Overextended restorations cannot be accurately assessed visually; hence, cross-sectional microscopy is essential for identifying the exact locations of preparation limits [30,31].

Our study used reflected light microscopy on section surfaces and micrometric techniques to assess the extent of filling deficits or excesses. The use of a layered restoration technique mitigates polymerization shrinkage effects and improves marginal adaptation, reducing marginal infiltration risks [12,32]. Achieving a perfect marginal adaptation close to 0 µm is nearly impossible, particularly in distal areas of the dental arches, such as wisdom teeth, due to anatomical constraints and limited visibility and access [32-35].

From the histogram, most maximum marginal adaptation errors fall within the 415-750 µm range. Achieving a marginal fit with an error magnitude below this value is challenging, even with magnification and precise handling of composite tools. Therefore, errors greater than 500 µm should be classified as clinical errors. The average marginal adaptation error in deficient areas was 364.3 µm, consistent with clinical experience indicating difficulty achieving sub-400 µm precision [20-22].

An error magnitude below 250 µm is ideal for marginal deficits, although challenging to achieve clinically, especially in distal arch areas. As illustrated in Figure 14, a 425 µm deficit affects marginal fit, potentially causing occlusal interferences. The cumulative frequency graph shows that approximately 20% of marginal adaptation errors are over 250 µm, with 15% considered clinical errors. About half of the errors are overextensions greater than 500 µm, indicating that overextension errors are twice as frequent as deficits.

The study's strengths include detailed microscopic analysis and comprehensive evaluation of restoration quality using both in vivo and laboratory stereomicroscopy. However, limitations include the sample size and challenges in collecting larger samples of extracted teeth with intact restorations due to periodontal disease and patient consent limitations. The findings, based on extracted teeth, may not fully represent in vivo conditions.

This study provides valuable insights into common errors in composite restorations and their clinical implications. While highlighting significant challenges, it points towards areas for improvement in clinical practice and the potential benefits of advanced microscopic techniques. Future research with larger sample sizes is recommended to validate these findings and enhance dental restoration quality.

### Strengths and limitations

Strengths of this study include the detailed microscopic analysis, which provided precise measurements of marginal adaptation errors. Using both in vivo and laboratory stereomicroscopy offered a comprehensive evaluation of restoration quality.

However, the study has limitations. The sample size may not be sufficient to generalize findings across all clinical settings. Additionally, collecting larger samples of extracted teeth with intact restorations is challenging due to the nature of periodontal disease and patient consent limitations. The findings are primarily based on extracted teeth, which may not fully represent in vivo conditions.

### Future recommendations

Future studies should aim to include larger and more diverse sample sizes to confirm these findings. Additional research could also explore the development of improved techniques and materials to reduce marginal adaptation errors, particularly in challenging distal areas of the dental arches. Continued advancements in microscopic evaluation methods will further enhance our understanding and management of composite restoration quality.

## CONCLUSION

This study underscores the significant challenges and errors associated with composite restorations, particularly in the posterior areas of the dental arches. Magnification in these regions is difficult, especially when coronal destruction involves the distal tooth surface. Achieving perfect marginal adaptation is nearly impossible, with errors frequently occurring as overextensions or deficits. Our findings reveal that overextensions are more common, with approximately half of the errors exceeding 500 µm. These are at least twice as frequent as deficits and can be classified as clinical errors. Deficits exceeding 250 µm, although less frequent, also constitute significant clinical errors. These results emphasize the importance of precise material handling and placement techniques to minimize these errors. The study also suggests that beveling enamel edges in occlusal cavities is not recommended due to the risk of thin restorative edges fracturing under occlusal forces. Marginal adaptation issues extend beyond the interface zone to include the cavity depth, necessitating comprehensive evaluation techniques like cross-sectional microscopy. While microscopic analysis offers detailed insights into restoration errors, achieving clinical perfection is challenging. The findings highlight the need for improved techniques and materials to enhance marginal adaptation, especially in distal areas of the dental arches. Future research should focus on strategies to reduce these errors and improve the overall quality of dental restorations.
